# Supplementary material for: Patterns of Intron Gain and Loss in Fungi
Source: PLoS Biol. 2004 Nov 30;2(12):e422. doi: 10.1371/journal.pbio.0020422 (PMC532390; doi:10.1371/journal.pbio.0020422)
Supplement: Table S1 — Also available at http://genes.mit.edu/NielsenEtAl/. (4.3 MB ZIP). [file pbio.0020422.st001.zip › NielsenEtAl/html/708.html]

AN1547.1.NCU09770.1.MG06609.1.FG08266.1


```
 CLUSTAL W (1.82) Multiple Sequence Alignments - Introns Inserted


Sequence 1: MG06609.1	526 aa
Sequence 2: FG08266.1	526 aa
Sequence 3: NCU09770.1	525 aa
Sequence 4: AN1547.1	525 aa
Alignment Length: 528 aa
Number Identitical Residues: 338 aa
Alignment Score (without introns) 15632


MG06609.1 	MASPVASAALKARIRSPPMLKKLCRPEDMLHHFPNGAYIGWSGFTGVGYPK2KTPTFLAD
NCU09770.1	MASPIASAALRARVKRPSMLKKLCNPEDMLQHFPNGAYIGWSGFTGVGYPK2KVPTMLAD
FG08266.1 	MASPVASAALKARVRNPALLKKLARPEDLMHHFPNGSYIGWSGFTAVGYPK2KVPAAMAD
AN1547.1  	MS---ASALLRSRVRRPSYLNKIAKAEDLIDLFPNGSYIGWSGFTGVGYPK2KVPTALAD
          	*:   *** *::*:: *. *:*:...**::. ****:********.***** *.*: :**

MG06609.1 	HVEKNNLQGKMKYSLFVGASSGTETEDRWASLDMINRRSPHQVGKNIAKGINSGKINFFD
NCU09770.1	HVEKNGLQGQLKYSLFVGASAGAETENRWAALDMIARRAPHQVGKNIAKGINEGRINFFD
FG08266.1 	YVEQNNLQSKMKYSLFVGASAGSETENRWAALDMINRRSPHQVGKDIAKGINSGRINFFD
AN1547.1  	HVEKNGLEGKLKYTLFVGASSGAETENRWARLNMIDRRSPHQVGKEIAKGINNGQIKFFD
          	:**:*.*:.::**:******:*:***:*** *:** **:******:******.*:*:***

MG06609.1 	KHLSMFPVDLVY0GFYTKDRPHNNLDVTVVEATEILEDGSIVPGASVGATPELIQMADK0
NCU09770.1	KHLSMFPVDLVY0GYYTKDRQNKNLDVVCVEATEIKEDGSIVLGASVGATPELIQMADK~
FG08266.1 	KHLSMFPSDLVY0GFYTKDRQNSNLDVTVVEATDILEDGSFVPGASVGATPELIQMADK0
AN1547.1  	KHLSMFPSDLTY0GWYTLNKPKNRIDVAVIEASAITEDGGIIPGASVGASPELVQMADK~
          	******* **.* *:** :: :..:**. :**: * ***.:: ******:***:***** 

MG06609.1 	IIIEVNTALPSMEGLHDITMTDLPPHRKPYLIQGVEDRIGTTSIPIDPSKVIGVIESDYQ
NCU09770.1	VIIEVNTAIPSFDGLHDITFSDLPPNRKPYLIQQCRDRIGTTSVPVDPEKVVGIIECTTP
FG08266.1 	VIIEVNTAIPSFKGLHDITFTDVPPHRTPYNITAVEDRIGSTSVKVDPSKVVAIVESDYW
AN1547.1  	VIIEVNTASPSFEGLHDITMSELPPNRKPYLILQPEDRIGTPHIPVDPEKVVAIVESDYP
          	:******* **:.******::::**:*.** *   .****:. : :**.**:.::*.   

MG06609.1 	DKTAPNTPADEGSAMIANHLIEYFEHEVKHGRMPKNLLPLQSGIGNIANAVIGGLAN--S
NCU09770.1	DQTLPNSPADETATAIAGHLIEFFEHEVAHGRLPKNLLPLQSGIGNIANAVIGGLET--S
FG08266.1 	DATGPNAAMDDTSRQIAGHLIEFFEHEVAHGRMPANLLPLQSGIGNVANAIVGGLNE--S
AN1547.1  	DQTQPNSPEDANSQAIASNLIEFLKHEVKHGRLPPNLLPIQSGIGNIANAVIGGLSKGGA
          	* * **:. *  :  **.:***:::*** ***:* ****:******:***::***  ..:

MG06609.1 	KFENLKVWTEVIQDTFLDLFDSGSLSYATATSVRFSPEGFERFYNNFDRYKERILLRSQS
NCU09770.1	NFKNLNVWTEVIQDTFLDLFDSGKLDFATATSIRFSPTGFERFYKNWDNYYDKLLLRSQS
FG08266.1 	KFKNLKVWTEVIQDTFLDLFDSGKLDYATATSTRFSPDGFKRFYDNWDAYADKILLRSQA
AN1547.1  	DFTNLKVWTEVLQDSFLDLFDSGNLDFATATSIRFSPDGFKRFYDNWERYAGKLLLRSQQ
          	.* **:*****:**:********.*.:***** **** **:***.*:: *  ::***** 

MG06609.1 	VSNAPEIIRRLGVIGMNTPVEVDIYAHANSTCVMGSRMLNGLGGSADFLRSAKYSIMHTP
NCU09770.1	VSNAPEIIRRLGVIGMNTPVEVDIYAHANSTNVMGSRMLNGLGGSADFLRNSKYSIMHTP
FG08266.1 	VSNAPEIIKRLGVIGMNTPVEVDIYAHANSTCVSGTRMLNGLGGSADFLRNAKYSIMHTP
AN1547.1  	VSNSPEIIRRLGCIGMNTPVEVDIYAHANSTCVMGSRMLNGLGGSADFLRSSKYSIMHTP
          	***:****:*** ****************** * *:**************.:********

MG06609.1 	STRPSKTDPHGVSCIVPMCTHIDQTEHDLDVVVTENGLADVRGLSPRERARVIIRQCAHP
NCU09770.1	STRPSKTDAHGVSCIVPMCTHVDQTEHDLDVIVTENGLADVRGLSPRERARVIIDKCAHD
FG08266.1 	SSRPSKTDPHGVSCIVPMCTHIDQTEHDLDVIVTEQGLADVRGLSPRERARVIINKCAHP
AN1547.1  	SVRPSKTDPTGVSCIVPFATHIDQTEHDLDVIVTEQGLADVRGLSPRERARVIIKKCSHP
          	* ******. *******:.**:*********:***:****************** :*:* 

MG06609.1 	VYRPILEAYFEKAEFECLRKGMGHEPHLLFNTFDMHKALQEEGSMQKVKSW
NCU09770.1	VYKPILKAYFEKAEFECLRKGMGHEPHLLFNSFDMHKALVEEGSMAKVKF-
FG08266.1 	KYQPILTHYYEKAESEGLKKGMGHEPHLLFNAFDLHKALAEEGSMLKVKPW
AN1547.1  	DYTPILTDYLDRAEFECLKKGMAHEPHLLFNAFKMHKNLQENGTMKISNWD
          	 * ***  * ::** * *:***.********:*.:** * *:*:*   :
```
